# Synthesis and characterization of magnetic nanoparticles coated with polystyrene sulfonic acid for biomedical applications

**DOI:** 10.1080/14686996.2020.1790032

**Published:** 2020-07-22

**Authors:** Bo-Wei Chen, Yun-Chi He, Shian-Ying Sung, Trang Thi Huynh Le, Chia-Ling Hsieh, Jiann-Yeu Chen, Zung-Hang Wei, Da-Jeng Yao

**Affiliations:** a Institute of NanoEngineering and MicroSystems, National Tsing Hua University, Hsinchu, Taiwan; b Department of Power Mechanical Engineering, National Tsing Hua University, Hsinchu, Taiwan; c Ph.D. Program for Translational Medicine, College of Medical Science and Technology, Taipei Medical University, Taipei, Taiwan; d TMU Research Center of Cancer Translational Medicine, Taipei Medical University, Taipei, Taiwan; e International Master/Ph.D. Program in Medicine, College of Medicine, Taipei Medical University, Taipei, Taiwan; f Center of Nanoscience and Nanotechnology, National Chung Hsing University, Taichung, Taiwan

**Keywords:** Magnetic nanoparticle, polystyrene sulfonic acid (PSS), superparamagnetic iron oxide (SPIO), biomedical applications, biocompatibility, 503 TEM, STEM, SEM; 203 Magnetics / Spintronics / Superconductors, 301 Chemical syntheses / processing, 504 X-ray / Neutron diffraction and scattering, 505 Optical / Molecular spectroscopy

## Abstract

The development of novel magnetic nanoparticles (MNPs) with satisfactory biocompatibility for biomedical applications has been the subject of extensive exploration over the past two decades. In this work, we synthesized superparamagnetic iron oxide MNPs coated with polystyrene sulfonic acid (PSS-MNPs) and with a conventional co-precipitation method. The core size and hydrodynamic diameter of the PSS-MNPs were determined as 8–18 nm and 50–200 nm with a transmission electron microscopy and dynamic light scattering, respectively. The saturation magnetization of the particles was measured as 60 emu g^−1^ with a superconducting quantum-interference-device magnetometer. The PSS content in the PSS-MNPs was 17% of the entire PSS-MNPs according to thermogravimetric analysis. Fourier-transform infrared spectra were recorded to detect the presence of SO_3_^−^ groups, which confirmed a successful PSS coating. The structural properties of the PSS-MNPs, including the crystalline lattice, composition and phases, were characterized with an X-ray powder diffractometer and 3D nanometer-scale Raman microspectrometer. MTT assay and Prussian-blue staining showed that, although PSS-MNPs caused no cytotoxicity in both NIH-3T3 mouse fibroblasts and SK-HEP1 human liver-cancer cells up to 1000 μg mL^−1^, SK-HEP1 cells exhibited significantly greater uptake of PSS-MNPs than NIH-3T3 cells. The low cytotoxicity and high biocompatibility of PSS-MNPs in human cancer cells demonstrated in the present work might have prospective applications for drug delivery.

## GRAPHICAL ABSTRACT

**Figure:**
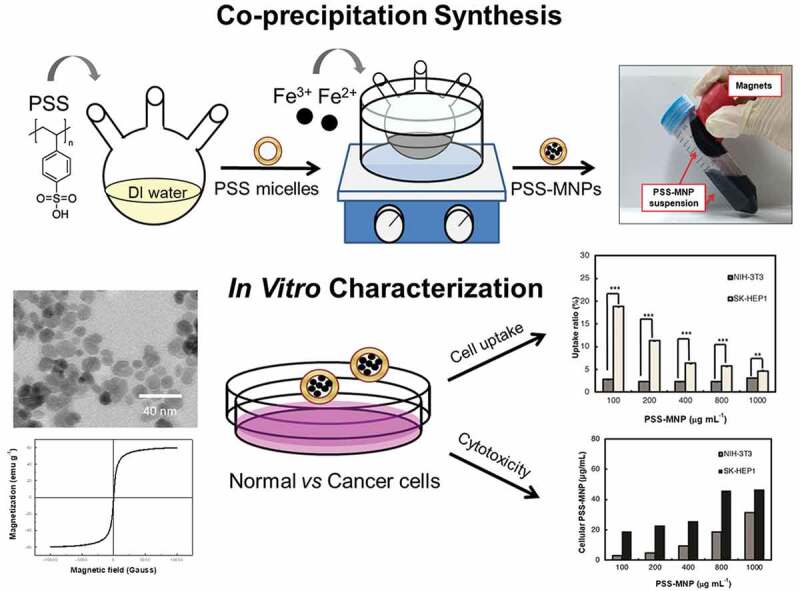

## 1. Introduction

Magnetic nanoparticles (MNPs) have attracted considerable attention for various biomedical applications [1–6], including nanocarriers for biochemical molecules or drug delivery, heat mediators in hyperthermia and contrast-imaging agents in magnetic-resonance imaging (MRI) and magnetic targeting. In these applications, MNPs of homogenous size and uniform shape are desired. The large ratios of surface to volume of MNPs have been known to cause extremely strong van der Waals interactions, which lead to the agglomeration of particles; this agglomeration cannot be uniformly distributed in a liquid phase, which becomes a major pitfall in biotechnological applications. Coating the MNPs with highly charged or sterically hindered molecules or polymers is a common method to overcome the attractive potential from the van der Waals effects; such a coating prevents agglomeration and increases the stability in aqueous media. Unfortunately, MNPs coated with charged molecules tend to aggregate readily in blood plasma [7]. Aggregated MNPs are recognized by the reticuloendothelial system and are consequently removed from the circulation [8]. The steric hindrance from the long-chain-like polymers provides a stable protective layer for the MNPs in a highly ionic solution and prevents agglomeration. With an excellent hydrophilic property, uncharged or weakly charged hydrophilic polymers, such as polyethylene glycol (PEG), polyvinylpyrrolidone (PVP), poly(lactic-co-glycolic acid) (PLGA), poly(vinyl alcohol) (PVA), chitosan and dextran (DEX) with high biocompatibility, have been widely used as protective shells to diminish the agglomeration of MNPs in the presence of serum proteins [9,10]. In addition to the protective shell, the magnetic core with ultra-small particle size and superparamagnetic properties has an effect to prevent agglomeration [10]. Superparamagnetic MNPs exhibit a zero average magnetization in the absence of an external magnetic field against agglomeration of MNPs caused by intermolecular magnetic moments and can be controlled and located at specific organs or tissues with an external magnetic field, offering a great prospect for drug delivery [11–13]. In recent years, more and more nanomedicines containing MNPs have been already approved by the U.S. Food and Drug Administration (FDA) for human use, mainly for in vivo administration [14]. Clearly, the toxicity of MNPs is one of the most debatable issues concerning the use. Much more effort is required to develop MNPs with improved biocompatible surface engineering to achieve minimal toxicity, for various applications in biomedicine.

Polystyrene sulfonic acid (PSS) is a hydrophilic chain-like polymer derived from polystyrene with SO_3_^−^ functional groups that has been reported for its antithrombotic and anticoagulant functions, as well as modulating activities of heparin-binding growth factors [15–19]. In aqueous applications, SO_3_^−^ groups are typically bonded or electrostatically attracted to an amino or diazo group and form covalent or non-covalent bonds between polymers [15,16,20]. In many cases, PSS can form polyelectrolyte multilayers or microcapsules in the presence of polyallylamine hydrochloride (PAH) with a layer-by-layer technique. The multilayers composed of PSS and PAH possess positive and negative charges in alternate layers; the films hence maintain structural stability in the absence of a chemical bond. Recent tests demonstrated that the multilayer PSS-PAH microcapsules can serve as fluorescent hydrophobic nanorods [21] or nanocarriers for drug doxorubicin [20]. PSS alone in the solvent can form micelles under vigorous stirring because of the hydrophilic group (-SO_3_H^−^) and hydrophobic alkyl group; it is therefore able to encapsulate nanomaterials and as-synthesized nuclei to form particles of size in a range of tens of nanometers [18]. Compared with polystyrene, which is one of the common polymers used for decorating MNPs, PSS possess stronger negative charge that can enhance both steric hindrance and electrostatic repulsions between nanoparticles when PSS is coated on the surface. Accordingly, PSS has an advantage over the uncharged or weakly charged hydrophilic polymers for nanoparticle biocompatibility by forming well-dispersed particles with narrow size distribution in the solvent whose properties closed to the biofluid of human body.

Co-precipitation synthesis procedure is the most widely used and a very simple method for the preparation of MNPs. In this method, the magnetic iron oxides are prepared from aqueous Fe^2+^ and Fe^3+^ salt solutions, by the addition of a base under an inert atmosphere at room temperatures or at high temperature. By varying the reaction conditions, such as the type of salts used, the ratio of ferric and ferrous ions, the PH value, the reaction temperature, and the other reaction parameters, magnetic phase and particle size of MNPs can be relatively broad [22,23]. With this method, our group has previously stabilized iron oxide MNPs by encapsulation with PSS to demonstrate the ability of concentric magnetic structures with a domain-wall pinning geometry in magnetic bead collection and magnetically labeled cell trapping [3]. Although the preparation of superparamagnetic MNPs coated with PSS (PSS-MNPs) has been well established [15], the applications and cell biocompatibility of PSS-MNPs are less investigated, in particular not at all in human cells. To further extend the possible biomedical application of PSS-MNPs in the area of oncology, in this work, we coated PSS on the surface of MNPs through a co-precipitation method with optimization and assessed the cell uptake and cytotoxicity of PSS-MNPs in cell culture model. The properties of synthetic PSS-MNPs, including size, crystal structures, composition and phases, were characterized with the appropriate instruments. The cell viability of both NIH-3T3 mouse normal fibroblasts and SK-HEP1 human hepatocellular carcinoma (HCC) cells was unaffected by treatment of PSS-MNPs at concentrations up to 1000 μg mL^−1^, indicating a suitable biosafety profile for biomedical applications. Notably, the rate of cellular uptake of PSS-MNPs in SK-HEP1 was significantly greater than that in NIH-3T3 cells. These results provided the first demonstration of excellent biocompatibility of PSS-MNPs in human cancer cells that implement PSS-MNPs for not only material engineering but also biological applications in the area of oncology.

## 2. Methods

### 2.1. Preparation of PSS-MNPs

For the preparation of PSS-MNPs, we refer to the reported co-precipitation method [16]. Briefly, 5 mL of 30% v/v PSS (molecular mass: 75,000, Sigma-Aldrich, USA) was added to 50 mL deionized water (DI water), and the temperature was increased to 65°C. To remove the oxygen from DI water, the solution was vigorously stirred (1100 rpm) with injection of gaseous nitrogen for 2 h. When PSS formed a large number micelles in the solution, 10 mL of 1 mM FeCl_3_ (iron [III] chloride, SHOWA, Japan) and 10 mL of 0.5 mM FeSO_4_ (iron [II] sulfate, SHOWA) were added consecutively. Subsequently, 7 mL of 5% v/v NH_4_OH (ammonia, Sigma Aldrich) was injected slowly with a peristaltic pump at rate 0.2 mL s^−1^. Following the injection, the solution turned from red to black. After 5 h, PSS-MNPs were washed several times with DI water to remove the excess NH_4_OH, uncoated PSS and possible ammonium chloride salts, and then collected with strong magnets for further characterization of their physical properties.

### 2.2. Thermogravimetric analysis (TGA)

To characterize the decomposition patterns of PSS-MNPs, TGA of the MNPs was measured with a thermal analyzer (Mettler-Toledo 2-HT, Switzerland) from 30°C to 1000°C at rate 10°C min^−1^ in an alumina pan (Al_2_O_3_) under a nitrogen atmosphere (flow rate: 35 mL min^−1^), to realize the composition and thermal stability of the coating. The MNPs were dried at 80°C to obtain 5 mg pure black powder before characterization. TGA calibration was checked by thermally degrading known weights of five metal standards, including In, Zn, Al, Au and Pd.

### 2.3. Attenuated-total-reflectance Fourier-transform infrared (ATR-FTIR) spectroscopy

ATR-FTIR spectroscopy was used to characterize PSS layer formed. IR spectra of PSS and PSS-MNPs were recorded with a Fourier-transform infrared spectrometer (Bruker Vertex 80 v, Germany) equipped with KBr beam splitter and DTGS-detector. The spectra of 20 mg of samples were recorded from 550 to 4000 cm^−1^ at room temperature at a resolution of 2 cm^−1^. The spectrometer was calibrated by running an OPUS Validation Program, a test using software for comprehensive instrument qualification, before performing.

### 2.4. Superconducting quantum-interference-device (SQUID) magnetometer

The magnetic properties of PSS-MNPs were measured with a SQUID magnetometer (Quantum Design, USA) at 300 K. Twenty mg of dried PSS-MNPs were characterized under an external magnetic field in a range of ±10,000 G. The calibration of SQUID before performing measurements was done with palladium reference sample at room temperature to adjust the correct magnetic moment.

### 2.5. X-ray powder diffraction (XRD)

To characterize the composition and structure of MNPs, 100 mg of dried PSS-MNPs were investigated with X-ray powder diffractometer (Cu Kα radiation, λ = 1.54 nm, Rigaku TTRAX III, Japan) for 2θ from 20° to 80° at a scan rate of 2° min^−1^ for the diffraction patterns and crystalline structure identification. Before sample characterization, the diffractometer was calibrated by loading a quartz standard slide and analyzing the quartz standard diffraction patterns and characteristic peaks through utilization of JADE software (Materials Data, Inc).

### 2.6. 3 D nanometer-scale Raman photoluminescence microspectrometer

To confirm the phase and identity of the MNPs after the XRD characterization, the samples (50 mg) were laminated and excited with 633 nm laser (He-Ne laser) in a 3D nanometer-scale Raman photoluminescence microspectrometer (Tokyo Instruments, Inc, Japan) to record the spectra in the range from 450 to 1000 cm^−1^. The wavelength of Raman spectrometer was calibrated through use of single crystal silicon wafer, of which Raman spectrum peak at 520 cm^−1^ under the configuration of 300 mm^−1^ grating, objective lens 100X (NA 0.9) and 1 second response time. The signals were recorded with a 20 second accumulation for 3 times to increase the overall signal-to-noise ratio.

### 2.7. Transmission electron microscope (TEM)

To determine the shape, size, and morphologies of the PSS-MNPs, a TEM (Philips field-emission, TECNAI 20, electron gun of ZrO/W(100) Schottky type, resolution ≤0.23 nm, Philips, Holland) was operated to obtain the images for the PSS-MNP morphology and core size. Before entry into the vacuum chamber, the PSS-MNPs were diluted with DI water into a concentration of <30 μg mL^−1^, casted on carbon-coated copper grids (200 mesh) and dried at 80°C for a few hours. TEM images were acquired using the TEM operated at accelerating voltage 80–200 kV.

### 2.8. Dynamic light-scattering (DLS) device

The hydrodynamic diameter of PSS-MNPs was evaluated in a DLS measurement (Brookhaven 90 plus particle-size analyzer, red diode laser, 40 mW, nominal wavelength 640 nm, Brookhaven Instruments Corp., USA) at 300 K. 25 mg PSS-MNPs were resuspended with 20 mL of DI water or phosphate buffered saline, and then 1 mL of solution was loaded in a quartz cuvette before operation of the DLS.

### 2.9. Cell culture

Human hepatoma cells SK-HEP-1 (ATCC® HTB-52TM) and mouse fibroblast cells NIH-3T3 (ATCC® CRL-1658™) were used; both cells were cultured following the standard cell-culture method^34^. All cell lines were grown in Dulbecco’s modified Eagle medium (DMEM, Life Technologies™) supplemented with 10% fetal bovine serum (FBS, Thermo Scientific, Fisher Scientific, USA) and 100 unit mL^−1^ penicillin/streptomycin (Thermo Scientific, Fisher Scientific, USA) and were incubated at 37°C in 5% CO_2_ atmosphere.

### 2.10. MTT assay

MTT assay is widely recognized as a reliable approach to detect cell viability. MTT assay involves the conversion of water-soluble 3-(4,5-dimethylthiazol-2-yl)-2,5-diphenyltetrazolium bromide into an insoluble formazan. The purple crystals of formazan become soluble in dimethyl sulfoxide (DMSO); the absorbance of the formazan solution is readable with an ELISA microplate reader at 570 nm. The concentration of formazan increased as the metabolically active cells used.

In this work, we prepared an aqueous solution of 3-(4,5-dimethylthiazol-2-yl)-2,5-diphenyltetrazolium bromide (concentration 2.5 g/50 g DI water, 5 wt%) as a MTT solution. The cells were seeded at a concentration 5 × 10^5^ cells mL^−1^ in culture dishes (35 mm) and were incubated in MTT solution (3 mL) at 37°C for 3 h. We subsequently removed the medium and replaced it with 1 mL of DMSO (100%). To quantify MTT-reactive cells, absorbance was measured on a microplate reader (Thermo Scientific, Fisher Scientific, USA). The assays were done in four replicates on four independent experiments.

### 2.11. Prussian-blue staining

A standard curve was generated from the measured absorbance of blue vehicles formed through the reaction between potassium ferrocyanide (K_4_[Fe(CN)_6_], SHOWA) and iron oxide product (EMG705, Ferrotec Corp.), at 620 nm with varied concentration. The values of concentration on the x-axis were converted into concentrations of PSS-MNPs assisted with the TGA results. Each data point represented a mean intensity integrated above the baseline and was quantified using the standard curve.

For quantification of PSS-MNP internalization, cells were seeded in a 96-well plate with a medium containing PSS-MNPs at 0, 100, 200, 400, 800 and 1000 μg mL^−1^ and were incubated at 37°C in 5% CO_2_ atmosphere for 24 h. After incubation, the cells were rinsed with phosphate buffered saline (PBS) to remove floating MNPs. The substances in each well were subsequently completely dissolved with 5 M hydrochloric acid (HCl, Sigma Aldrich) solution at 65°C for 2 h; thereafter, 2% K_4_[Fe(CN)_6_] solution was added to digest the substance; gentle boiling was performed until a clear blue solution was obtained. A comparison of measured absorbance values with the standard curve was performed to calculate and to evaluate the PSS-MNP uptake ratio on converting the absorbance values.

### 2.12. Statistical analysis

The values acquired from a population of cells are represented as mean ± standard deviation (SD). At least three independent tests were performed to obtain the final results. Student’s *t* test was used to compare cell viability between untreated PSS-MNPs and treated PSS-MNPs cell groups of distinct cell types; the significance level was set at *p* < 0.05, *p* < 0.01 and *p* < 0.001.

## 3. Results

### 3.1. Physical characterization of PSS-MNPs

**Figure 1.**
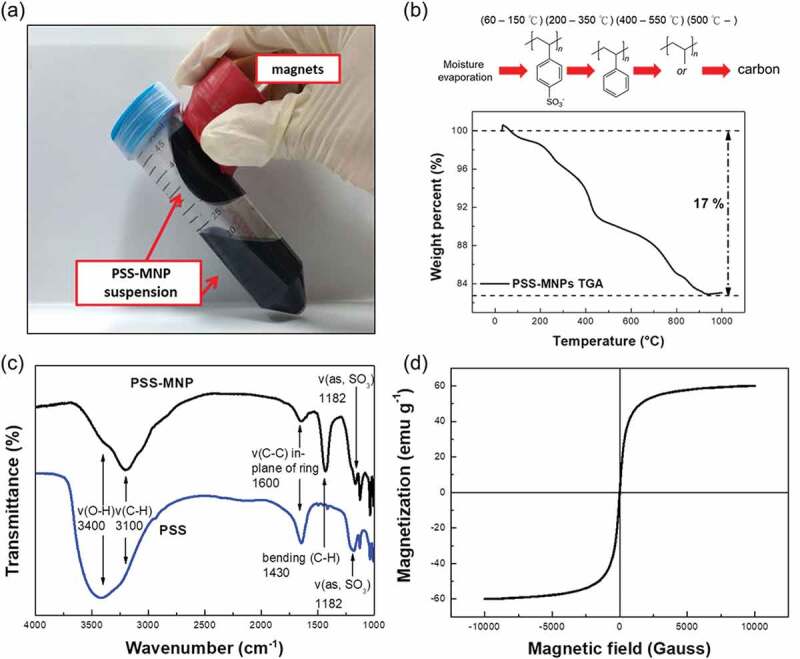
Image of PSS-MNPs attracted with a strong rare-earth magnet, thermal analyses, FTIR spectra and magnetization curve of PSS-MNPs. (a) The suspension stability of PSS-MNPs is clear from the lack of precipitation and aggregation toward the magnet. (b) Thermal analyses of PSS-MNPs under the nitrogen atmosphere from room temperature to 1000°C. The content of coated polymer on the MNPs was 17% of the whole PSS-MNPs. (c) FTIR spectra of PSS molecules and PSS-MNPs. The FTIR instrument was operated in air at 300 K. (d) Magnetization curve of PSS-MNPs measured with a SQUID magnetometer in vacuum at 300 K.

The suspension stability of MNPs is the major obstacle to their biotechnological applications. In this work, we synthesized PSS-coated Fe_3_O_4_ using a co-precipitation method based on our previous established protocols [3] with modification of a fixed reaction temperature of 65°C and a relatively slow dropping speed of basic solution of 0.2 mL s^−1^ (for more details see Methods section). Unlike bare MNPs, the PSS-MNPs showed no precipitation and remained uniformly distributed as a pure-black solution instead of an aggregation of bare MNPs that separated from the deionized water (Figure 1(a)). We were also able to move the entire PSS-MNPs solution in the tube on applying an external magnetic field, indicating that the enhanced dispersion of PSS-MNPs in water is due to the PSS coating. The TGA result for PSS-MNPs (Figure 1(b)) exhibited three clear steps of weight loss in the studied temperature range. The first stage represented an endothermic peak between 60°C and 150°C, which involved dehydration and removal of the moisture on the MNPs due to the PSS hydrophilicity. An intense destruction of PSS-MNPs was observed in the second (main) stage at higher temperature that was associated with the desulfonation of sulfonic-acid groups (SO_3_ H) and the decomposition of polystyrene in the range 200–550°C [24,25]; the main carbon-chain scission extended to about 900°C [26]. In this temperature range, a mass loss of 17% was observed and attributed to the loss of PSS.

To confirm whether the thin transparent membrane was composed of PSS, we applied an FTIR spectrometer to record the absorbance spectra of samples in the range 1000–4000 cm^−1^ to detect the vibrational modes of bonds. The composition of the coating layer on the MNPs was further identified through the analysis of characteristic peaks at various wavenumbers. Figure 1(c) presents the FTIR spectra of PSS molecules and PSS-MNPs. The presence of PSS was observed at the characteristic bands of 1182 cm^−1^ and 1600 cm^−1^ near the asymmetric stretch of S = O bonds and the vibration of the C-C bond of benzene rings, respectively. These two major peaks proved that PSS was successfully coated on the MNPs [27]. A SQUID (MPMS5, Quantum Design, USA) was used to evaluate the magnetic properties of PSS-MNPs at 300 K. Figure 1(d) shows the superparamagnetic magnetization curve of PSS-MNPs in the absence of hysteresis, for which the saturation magnetization was 60 emu g^−1^.

**Figure 2.**
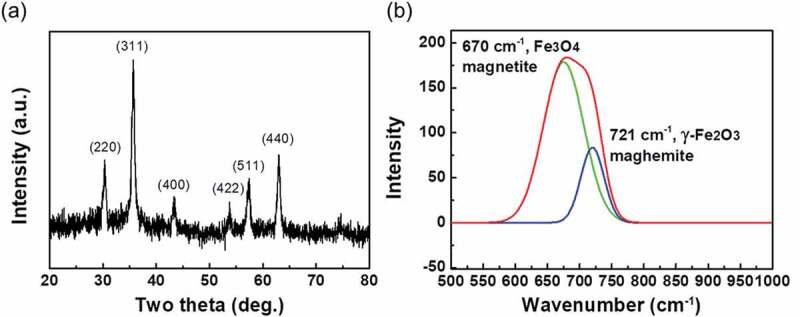
XRD pattern and Raman spectrum of PSS-MNPs. (a) Characteristic peaks in the XRD pattern are located at 30.1°, 35.5°, 43.1°, 53.4°, 57.0° and 62.6° for (220), (311), (400), (422), (511) and (440) planes of Fe_3_O_4_ and γ-Fe_2_O_3_, respectively. (b) For the characteristics of PSS-MNPs (red line) the Raman spectrum shows two lines at 670 cm^−1^ (green line) and 721 cm^−1^ (blue line) for Fe_3_O_4_ and γ-Fe_2_O_3_, respectively.

To identify the composition and face-centered cubic crystal structure of PSS-MNPs, we applied XRD with Bragg’s law to obtain the parameters of the unit lattice, which determine the crystalline composition. Figure 2(a) displays the XRD pattern of the PSS-MNPs. Characteristic signals are located at 30.1°, 35.5°, 43.1°, 53.4°, 57.0° and 62.6°, representing the (220), (311), (400), (422), (511) and (440) planes, respectively, showing that iron oxide was formed in mostly the magnetite (Fe_3_O_4_) and maghemite (γ-Fe_2_O_3_) phases [28].

As Fe_3_O_4_ and γ-Fe_2_O_3_ have same crystal structure, it is difficult to distinguish between them with XRD. A Raman spectrum was recorded to identify the phases of iron oxide present in the MNPs. As shown in Figure 2(b), an intense band in the Raman spectrum of the PSS-MNPs was observed in the region 600–760 cm^−1^, which is considered to indicate the presence of magnetite (670 cm^−1^) mixed with maghemite (721 cm^−1^). Taken together, these results verified that PSS-MNPs were composed of both Fe_3_O_4_ andγ-Fe_2_O_3_ phases in the structures [29].

**Figure 3.**
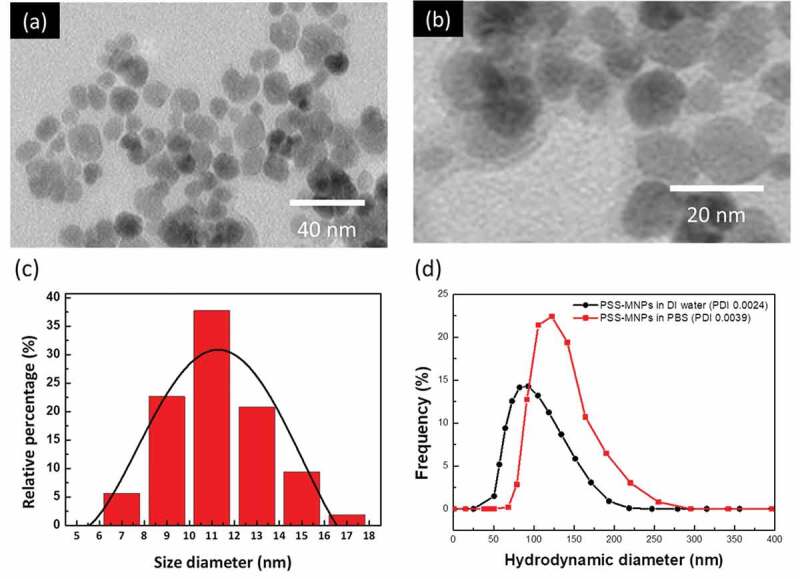
TEM images and DLS analysis of MNPs coated with PSS. (a) TEM images of MNPs coated with PSS; scale bar 40 nm, (b) enlarged TEM image, scale bar 20 nm, (c) size distribution estimated from TEM images of (a), and (d) hydrodynamic diameter (with PDI) in the DLS measurement of PSS-MNPs. *PDI = polydispersity index.

The particle size and hydrodynamic diameter of PSS-MNPs are key factors that enable the endocytosis of MNPs into cells [17]. Both TEM and DLS were used to determine the morphology, particle size and hydrodynamic radius. As shown in Figure 3(a,b) (enlarged version), the TEM image demonstrated that a thin transparent membrane was coated on MNPs; there was no evidence of agglomeration of PSS-MNPs. The transparent membrane is predicted to be PSS molecules since there was no component other than PSS can form micelles encapsulating ion oxides in the reaction of co-precipitation. The distribution of core diameter (Figure 3(c)) ranged between 8 and 18 nm with an average size of 11.3 nm. Moreover, the hydrodynamic diameter of the PSS-MNPs ranged between 50 and 200 nm (average hydrodynamic diameter 99 nm) at pH 6.6 in water (Figure 3(d)), for which the polydispersity index (PDI) was 0.0024, showing the monodispersity of the formed MNPs. Unlike TEM that provides primary particle size in dried form, DLS measures the hydrodynamic diameter of the particles in liquid, which includes not just the particle itself, but the polymer shells or the ionic and solvent layers associated with it in solution [30], leading to a larger particle size than that measured by TEM.

To further characterize the disperse phase of PSS-MNPs under physiological condition, additional DLS analysis in an aqueous solution of PBS was conducted. Although hydrodynamic diameter (75–250 nm; an average hydrodynamic diameter peak at 130 nm) and PDI (0.0039) in PBS were increased compared to that in DI water (Figure 3(b)), the value was still characteristic of monodispersity.

### 3.2. Cellular internalization and cytotoxicity of PSS-MNPs

To assess the feasibility of PSS-MNPs for future medical applications such as labeling and detection of cancer cells, we selected a HCC cell line SK-HEP1, because HCC is a common malignant disease and the second leading cause of cancer death in Taiwan [31].

The availability of primary hepatocytes is limited, especially human primary hepatocytes. Alternatively, the NIH-3T3 fibroblast cell line that is frequently used as a normal cell control in the field of cancer research was included for comparison [32].

**Figure 4.**
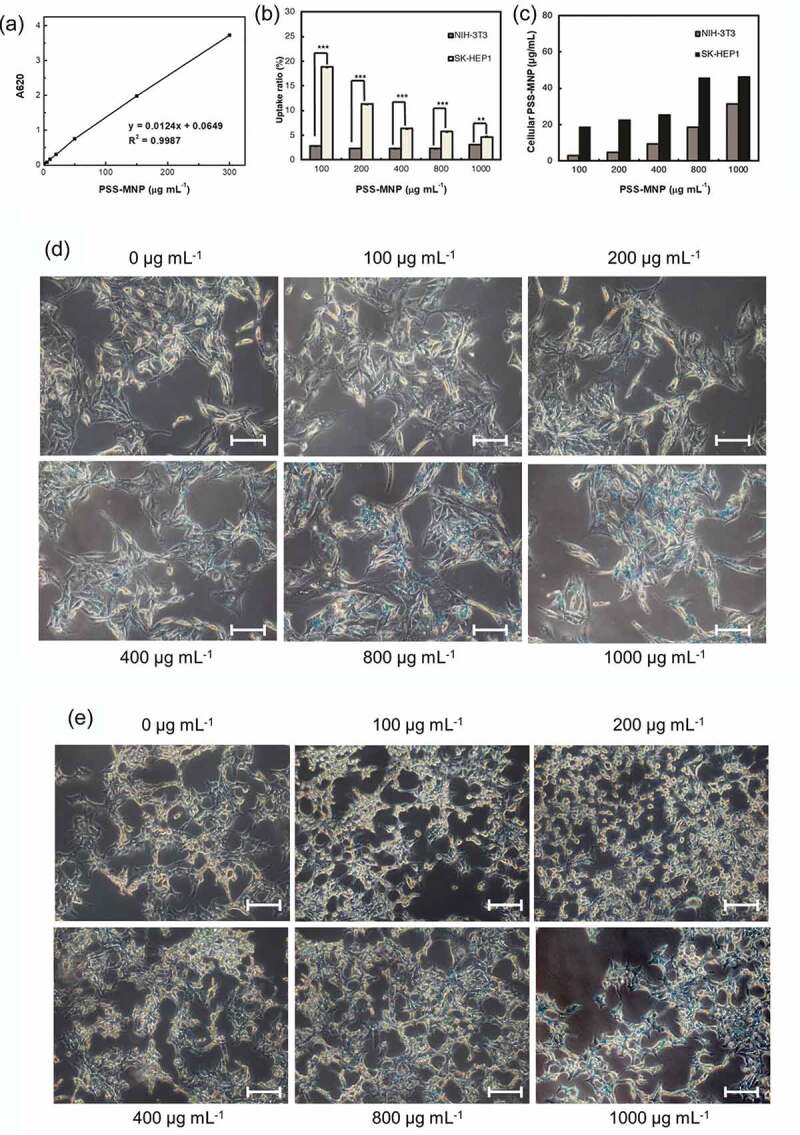
Quantification and observation of PSS-MNP internalization into NIH-3T3 and SK-HEP1 cells. (a) Standard curve for analysis of Fe_3_O_4_ through measured absorption at 620 nm (A620). *R*^2^: accuracy of standard curve. (b, c) Comparison of cellular uptake of PSS-MNPs at concentrations 100, 200, 400, 800, 1000 μg mL^−1^ between NIH-3T3 and SK-HEP1 cell lines; the amount of cellular PSS-MNPs was quantified with the standard curve. The data are presented as uptake ratio (b) and absolute concentration (c) as mean ± SD of 3 independent experiments (*n* = 6). The degree of significance is given as ** *p* < 0.01 and *** *p* < 0.001. Prussian-blue staining of (d) NIH-3T3 and (e) SK-HEP1 cells treated with PSS-MNPs. The experiment was performed in duplicate; a representative image (20× objective) is shown. Scale bar = 30 µm.

We first characterized the efficiency of the cellular internalization of PSS-MNPs; SK-HEP1 and NIH-3T3 cells were incubated with a medium containing PSS-MNPs at 100, 200, 400, 800 and 1000 μg mL^−1^ for 24 h, then subjected to Prussian-blue staining followed by absorbance measurements at 620 nm with a microplate reader (Thermo Scientific, Fisher Scientific, USA). The amount of intracellular PSS-MNPs in each treatment condition was calculated based on a standard curve (Figure 4(a)). Quantification of the uptake confirmed a dose-dependent increase of intracellular amount of PSS-MNPs in both cell lines with a significantly greater amount in the SK-HEP1 group than in the NIH-3T3. Notably, although the rate of PSS-MNP uptake remained roughly constant at approximately 3% under varied concentrations of the PSS-MNP treatment in NIH-3T3 cells, a maximal rate 18.8% of uptake was observed at concentration 100 μg mL^−1^ and decreased with increasing concentration of PSS-MNPs in SK-HEP1 (Figure 4(b,c)). Prussian-blue staining revealed the presence of distributed iron-containing granules (stained blue) in these cells receiving PSS-MNP treatment (Figure 4(d,e)). The majority of the blue granules can be seen inside the cytoplasm around the nuclei of cells (supplementary Figure S1), verifying the cellular internalization and distribution of the PSS-MNPs.

**Figure 5.**
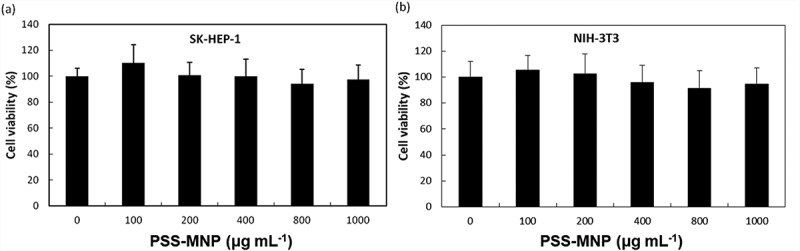
Cytotoxicity of PSS-MNPs in (a) SK-HEP1 and (b) NIH-3T3 cells. Cells were treated with PSS-MNPs at concentrations 100, 200, 400, 800 and 1000 μg mL^−1^ through a MTT assay.

Toxicity issues are important factors in the context of a biomaterial. To assess whether treatment with PSS-MMP affects the viability of cells, we incubated SK-HEP1 and NIH-3T3 cells with PSS-MMP at increasing concentrations for 24 h and analyzed the cell viability with a MTT assay. As Figure 5 shows, both SK-HEP1 (Figure 5(a)) and NIH-3T3 (Figure 5(b)) cells that were treated with PSS-MNPs exhibited no significantly reduced viability up to concentration 1000 μg mL^−1^ tested by comparison with untreated control cultures. By contrast, while treatment with commercialized water-based MNPs (EMG 705) that was used as the uncoated MNP control resulted in a > 40% reduction in viability in both cell lines starting at the doses of 150 μg mL^−1^, the cell uptake of EMG 705 remained significantly lower than that of PSS-MNPs (Supplementary Figure S2). These results demonstrate remarkable biocompatibility of PSS-MNPs for cells.

## 4. Discussion

For applications *in vivo*, MNPs are required to be superparamagnetic in nature and to have a surface functionalized to enhance their biocompatibility, blood circulation period and stability. In the present work, we synthesized PSS-MNPs comprising magnetic iron oxides coated with polymer PSS and defined their characteristics as follows. (1) The size of the iron oxide core was between 8 and 18 nm (Figure 3(c)) with an average hydrodynamic diameter of 99 nm (coating included) (Figure 3(d)), which is in the range of the superparamagnetic limit as well as mediating passive tumor extravasation [33–35]. (2) The PDI value 0.0039 is smaller than 0.15, a feature indicating the presence of unaggregated monodisperse nanoparticles [36], an essential factor for a nanodelivery system [37]. (3) The approximately 6-fold higher cellular uptake rate of PSS-MNPs in SK-HEP1 tumor cells than NIH-3T3 normal cells occurred under the low concentration (Figure 4), indicating easy access to tumor target. (4) Both human and mouse cells remained viable following exposure to PSS-MNPs (1 mg mL^−1^) for 24 h (Figure 5), implying that the nanoparticles were well tolerated by living cells and exhibit an apparently small toxicity for various biological applications. These characteristics are relevant for the rational design and subsequent utilization of PSS-MNPs for biomedical applications, for detection and imaging of human cancer and for cancer-targeted therapy with hyperthermia and releasing anti-cancer molecules with significantly reduced side effects.

The properties of nanoparticles depend mainly on the nature of their synthesis. Co-precipitation and thermal decomposition are currently the most common chemical methods to obtain MNPs for various applications notably in nanobiomedicine, due to their potential to achieve large quantity and desired size, morphology, structure and magnetic control [38]. Although thermal decomposition can produce MNPs of greater quality than co-precipitation, the requirement of temperature in a range between 200°C and 300°C for reaction and the as-prepared MNPs only dispersible in organic solvents limit its applications in the field of biology. To avoid any desulfonation reaction [20] occurring during the production procedure and to retain the biocompatibility of nanoparticles, we used co-precipitation synthesis to prepare PSS-MNPs with temperature less than 70°C and DI water as solvent. With a modification that prolonged the stirring duration of PSS in DI water under bubbled nitrogen gas to create an oxygen-free environment before adding iron precursors, we obtained monodisperse MNPs with extremely high magnetic properties, achieving the saturation magnetization value 60 emu g^−1^ Fe_3_O_4_. During this process, PSS micelles acted as a coating material and concurrently served as a shell layer to protect the magnetite from oxidation to maghemite.

Many critical in vivo functions of nanoparticles, such as circulation period, internalization and clearance have been proved to depend on their size. Several authors have indicated that nanoparticles of diameter greater than 200 nm activate the complement system and become rapidly removed from a blood stream, accumulating in the liver and spleen; particles of diameter less than approximately 10 nm are also rapidly eliminated by the kidneys [39].

Previous work that evaluated the effect of particle size on cellular uptake of polymeric nanoparticles showed that nanoparticles of size 100 nm acquired the best properties for cellular uptake, among sizes 50, 200, 500 and 1000 nm [40]. Despite the small size (11.3 nm) of the iron core, the hydrodynamic diameter of PSS-MNPs in a range between 50 and 200 nm conforms well with the effective size cutoff for long circulation retention and cellular uptake.

To compare the internalization of PSS-MNPs between normal and tumor cells, we used NIH-3T3 fibroblasts and SK-HEP1 HCC cells as a proof-of-principle demonstration. Our results of the quantification of MNPs uptaken by cells (Figure 4) provide evidence that PSS-MNPs are more accessible in tumor cells, which might benefit from targeting of cancers. Although the mechanism underlying the preferential internalization of PSS-MNPs into the SK-HEP1 cells remains to be determined, the nanoparticle size and surface coating have been known to be key determinants of the uptake pathways [41,42]. Other than particle size, it is, in general, assumed that anionic nanoparticles appear to be mainly taken up by caveolae-mediated endocytosis. Caveolae are plasma membrane invaginations with size in a range typically from 50 to 100 nm and are composed of membrane protein caveolin, which confers on them a flask-shaped structure. Among three known caveolins (CAV-1, 2 and 3), caveolin-1 (CAV-1) is ubiquitously expressed in all cell types and was found to be over-expressed in numerous cancers in which it can act as a positive regulator of stress resistance and survival [43]. A recent meta-analytical study [44] supported that CAV-1 is correlated with an unfavorable clinico-pathological status of HCC, including a low degree of differentiation and metastasis. Based on the particle size and surface charge, the high rate of PSS-MNP internalization in SK-HEP1 HCC cells occurs, perhaps, through the enhanced caveolae-mediated endocytosis pathway that is contributed by the over-expressed CAV-1 on the cells. This feature in combination with greater saturation magnetization 60 emu g^−1^ Fe_3_O_4_ makes the present PSS-MNPs superior to previously reported MNPs coated with polymers of other types, including PEG [45–47], DEX [47–49], PVP [50] and PVA [51] for HCC theranostics with an applied magnetic field. We are currently optimizing the protocol of enhanced stability and cargo encapsulation. With these improvements, a further assessment of PSS-MNP-mediated drug delivery or hyperthermia for the treatment of HCC is thereby warranted.

## 5. Conclusions

In this study, we synthesized PSS-MNPs through a modified co-precipitation method to improve cell biocompatibility. The PSS molecules were successfully coated on the surface of MNPs, as revealed by ATR-FTIR spectroscopy. From the subsequent data acquired from TEM, DLS, SQUID, XRD and Raman spectroscopy, the PSS-MNPs were confirmed to be monodisperse and superparamagnetic and consist of Fe_3_O_4_ and γ-Fe_2_O_3_. The greater cellular uptake of PSS-MNPs for HCC SK-HEP1 than that for normal NIH-3T3 cells was proved by Prussian blue staining, and it did not cause a significant reduction in cell viability. Taken together, we have synthesized and characterized PSS-MNPs as a novel, superparamagnetic and biocompatible material that has a potential application as magnetic carriers for HCC detection or treatment.

## Supplementary Material

Supplemental Material
