# Supplementary material for: Synthesis and characterization of magnetic nanoparticles coated with polystyrene sulfonic acid for biomedical applications
Source: Sci Technol Adv Mater. 2020 Jul 22;21(1):471–81. doi: 10.1080/14686996.2020.1790032 (PMC7476547; doi:10.1080/14686996.2020.1790032)
Supplement: Supplemental Material [file TSTA_A_1790032_SM0187.pdf]

Synthesis and Characterization of Magnetic Nanoparticles coated with Polystyrene Sulfonic Acid for Biomedical Applications

Bo-Wei Chen[1#], Yun-Chi He[2#], Shian-Ying Sung[3,4], Trang Thi Huynh Le[5], Chia-Ling Hsieh[3,4*], Jiann-Yeu Chen[6], Zung-Hang Wei[1,2] and Da-Jeng Yao[1,2*]

1 Institute of NanoEngineering and MicroSystems, National Tsing Hua University, Hsinchu, Taiwan

2 Department of Power Mechanical Engineering, National Tsing Hua University, Hsinchu, Taiwan

3 Ph.D. Program for Translational Medicine, College of Medical Science and Technology, Taipei Medical University, Taipei, Taiwan

4 TMU Research Center of Cancer Translational Medicine, Taipei Medical University, Taipei, Taiwan

5 International Master/Ph.D. Program in Medicine, College of Medicine, Taipei Medical University, Taipei, Taiwan

6 Center of Nanoscience and Nanotechnology, National Chung Hsing University, Taichung, Taiwan

*Correspondence and requests for materials should be addressed to C.-L.H. (email: chsieh2@tmu.edu.tw) and D.-J.Y. (djyao@mx.nthu.edu.tw)

# These authors contributed equally to this work

NIH 3T3 SK-HEP1

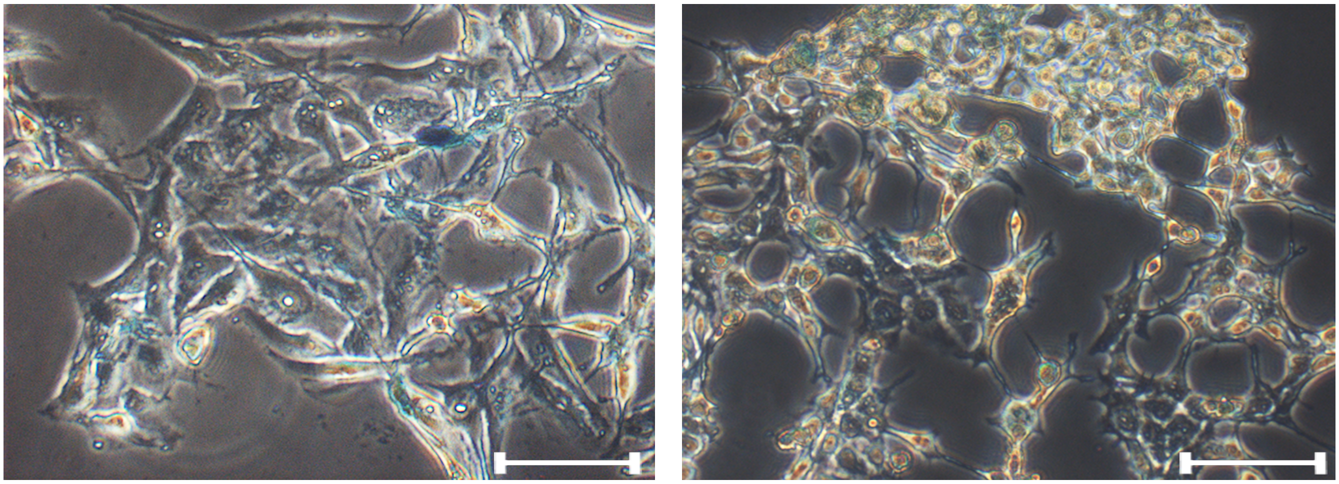

Figure S1. Prussian blue staining for PSS-MNPs-positive cells. The cropped images of Figures 4d,e (400 μg mL^{-1}) shows the cellular locazation of PSS-MNPs at the cytoplasm around the nuclei of cells. Scale bar = 30 μm.

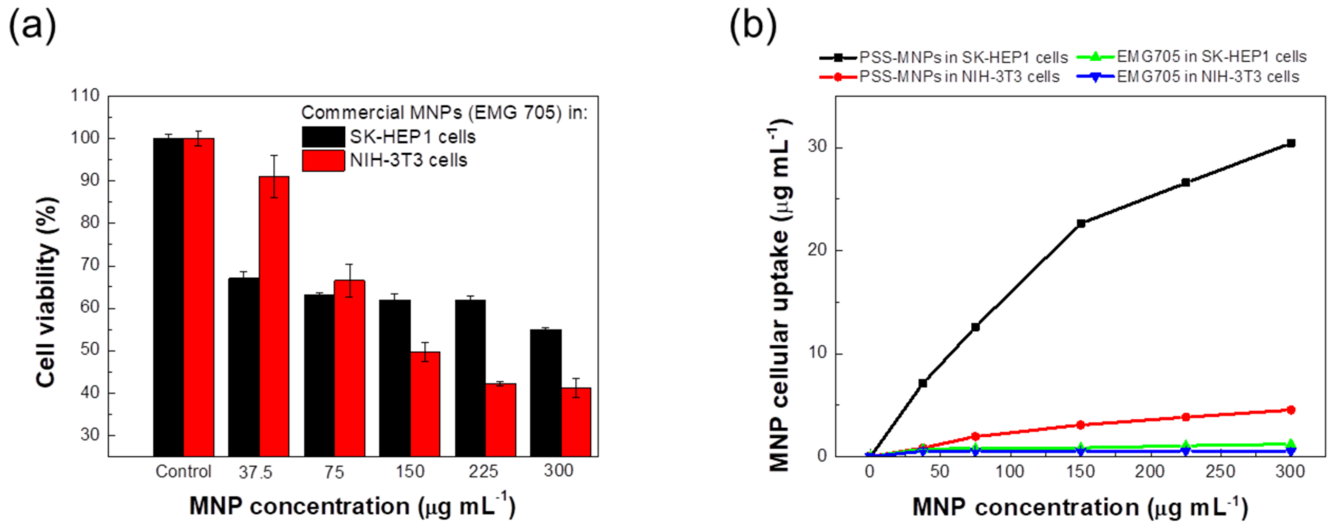

Figure S2. Cytotoxicity and cellular uptake of a commercialized MNP product EMG 705 (Ferrotec Corp.) in SK-HEP1 and NIH-3T3 cells. Cells were treated with EMG 705 at concentrations of 37.5, 75, 150 and 300 μg mL^{-1} for 24 h and then subjected to (a) MTT cell viability assay and (b) Prussian blue assay for MNP contents. The data is presented as as mean ± SD of 3 independent experiments (n = 6).
